# HS-SPME-GC-MS Analyses of Volatiles in Plant Populations—Quantitating Compound × Individual Matrix Effects

**DOI:** 10.3390/molecules23102436

**Published:** 2018-09-23

**Authors:** Elizabeth A. Burzynski-Chang, Imelda Ryona, Bruce I. Reisch, Itay Gonda, Majid R. Foolad, James J. Giovannoni, Gavin L. Sacks

**Affiliations:** 1 Department of Food Science, Stocking Hall, Cornell University, Ithaca, NY 14853, USA; eab54@cornell.edu (E.A.B.-C.); ir45@cornell.edu (I.R.); 2 Horticulture Section, School of Integrative Plant Science, New York State Agricultural Experiment Station, Cornell University, Geneva, NY 14456, USA; bir1@cornell.edu; 3 Boyce Thompson Institute for Plant Science, Ithaca, NY 14853, USA; itaygonda@gmail.com (I.G.); james.giovannoni@ars.usda.gov (J.J.G.); 4 Department of Plant Science, Pennsylvania State University, University Park, PA 16802, USA; mrf5@psu.edu

**Keywords:** breeding population, internal standards, matrix effects, plant volatiles, SPME, odorant analysis

## Abstract

Headspace solid-phase microextraction (HS-SPME) coupled to gas chromatography–mass spectrometry (GC-MS) is widely employed for volatile analyses of plants, including mapping populations used in plant breeding research. Studies often employ a single internal surrogate standard, even when multiple analytes are measured, with the assumption that any relative changes in matrix effects among individuals would be similar for all compounds, i.e., matrix effects do not show Compound × Individual interactions. We tested this assumption using individuals from two plant populations: an interspecific grape (*Vitis* spp.) mapping population (*n* = 140) and a tomato (*Solanum* spp.) recombinant inbred line (RIL) population (*n* = 148). Individual plants from the two populations were spiked with a cocktail of internal standards (*n* = 6, 9, respectively) prior to HS-SPME-GC-MS. Variation in the relative responses of internal standards indicated that Compound × Individual interactions exist but were different between the two populations. For the grape population, relative responses among pairs of internal standards varied considerably among individuals, with a maximum of 249% relative standard deviation (RSD) for the pair of [U^13^C]hexanal and [U^13^C]hexanol. However, in the tomato population, relative responses of internal standard pairs varied much less, with pairwise RSDs ranging from 8% to 56%. The approach described in this paper could be used to evaluate the suitability of using surrogate standards for HS-SPME-GC-MS studies in other plant populations.

## 1. Introduction

Headspace solid-phase microextraction (HS-SPME) is widely employed to isolate and pre-concentrate volatiles prior to gas chromatography–mass spectrometry (GC-MS) analysis [1,2,3,4]. SPME has several advantages over other sample preparation techniques (e.g., solid-phase extraction or liquid–liquid extraction), including its avoidance of solvents, ease of automation, and small sample size requirements [5]. These features make SPME particularly well suited for studies that require analysis of a large number of samples, e.g., when evaluating plant populations used by breeders in investigating the genetic underpinnings of traits [6,7]. Mapping of genes or quantitative trait loci (QTLs) controlling volatiles—including those associated with aroma—has been reported in several plant species, including tomatoes [2], melons [8,9], apples [10,11], and grapes [12,13,14]. Plant volatile phenotyping is usually performed by GC-MS, for both targeted analyses of a small number of volatiles [15,16] and broader profiling of a large number of targeted or nontargeted volatiles (“metabolomics”) [1,2,8,9,10,17].

A challenge associated with SPME, however, is its high susceptibility to matrix effects, including plant matrixes [18,19]. For example, for a range of volatiles, SPME-GC-MS responses were reported to decrease by 2- to 12-fold in a tomato matrix [15]. This decrease could arise from either competition on the SPME fiber or decreases in analyte volatility. Matrix effects could be compensated for through appropriate calibration, most commonly through the use of well-matched and equilibrated internal standards. When available, the preferred choice for an internal standard is a stable isotope-labeled analogue of the target analyte, i.e., stable isotope dilution analysis (SIDA) [20]. This technique has been employed in grape mapping populations to identify candidate genes associated with monoterpene production (“muscat” aroma) [12] following a solid-phase extraction, and in basmati rice grains for phenotype 2-acetyl-1-pyrroline (“nutty” aroma) following SPME [21]. SIDA, however, is not employed in most volatile phenotyping studies of breeding populations, including those using SPME, likely due to the high cost or commercial unavailability of isotopically labeled standards [20]. The impracticality of SIDA is particularly severe for nontargeted studies, which may involve measurement of dozens of volatiles whose identity is unknown prior to analysis. Instead, it is common for SPME-based volatile phenotyping studies to use a single surrogate standard or to normalize responses to the total ion count. In this approach, it is assumed that the relative matrix effects on any given compound (analyte or standard) are consistent among individuals, i.e., relative differences in analyte concentrations are preserved. Knowledge of relative ratios of volatiles is still potentially useful, e.g., in QTL analyses to identify associated genetic markers for breeding purposes or identify likely metabolic networks. However, the assumption that matrix effects do not show Compound × Individual interactions in ostensibly similar samples is not always valid. For example, the relative response for an *n*-decane surrogate in soybean oil changed by up to 8-fold as compared to ^13^C-labeled internal standards following thermal oxidation of the oil matrix [22]. Similarly, modest variations in ethanol content of model wines caused Compound × Individual matrix effects across a range of volatiles [23]. These effects would not allow for accurate relative quantification based on a single surrogate standard.

Although the occurrence of Compound × Individual matrix effects during SPME analysis of plant populations can be assumed, the extent of such interactions has not been quantitated and routine approaches to their determination have not been described. Evaluating the extent of these interactions is becoming more important in plant breeding research due to a greater interest in improving fruit flavor, in comparison to the historic focus of plant breeding on improving yield, storage characteristics, and disease resistance [24]. Compound × Individual interactions could potentially be much smaller than variation from other sources, e.g., biological variability, in which case the error introduced from using a surrogate standard would be tolerable. To our knowledge, an approach to quantitate the extent of Compound × Individual matrix effects during HS-SPME-GC-MS analyses—including analyses of plant populations—has not been described, even though this phenomenon is well known among analytical chemists to exist [22,25]. We hypothesized that these effects could be evaluated by comparing the relative responses of multiple internal standards within a population. In this report, we describe our approach, and use it to evaluate the extent of Compound × Individual matrix effects in a grape mapping population and a tomato recombinant inbred line (RIL) population. 

## 2. Results and Discussion

### 2.1. General Approach to Estimating Compound × Sample Interactions ($\sigma_{std\;1,std\;2}$) in Plant Populations

**Figure 1:**
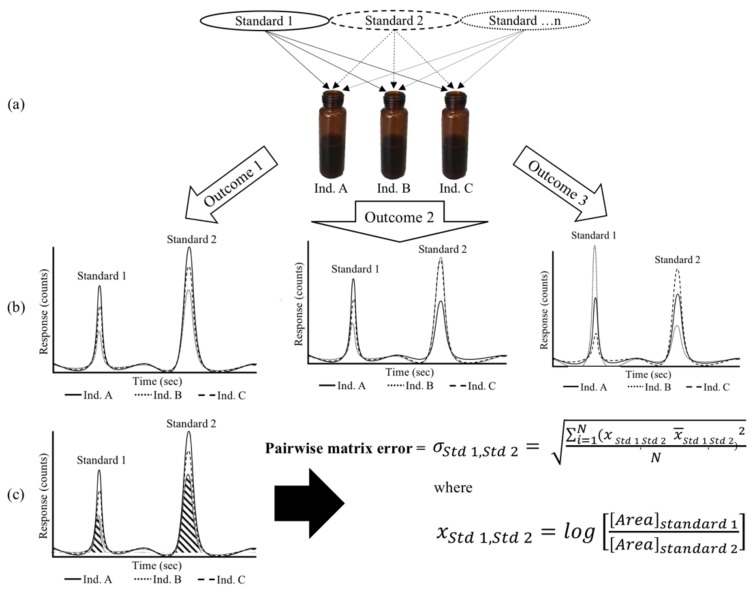
Overview of the experimental design. (**a**) Multiple non-native internal standards were added to each plant individual during sample preparation. (**b**) Samples were analyzed by HS-SPME-GC-MS (simulated data shown). In some cases, pairs of standards had similar relative ratios across multiple individuals (Outcome 1), while in other cases there was evidence of Compound × Individual matrix effects, in which the relative peak areas for pairs of standards changed among individuals (Outcomes 2 and 3). (**c**) Compound × Individual matrix effects were quantitated by comparing the log-transformed integrated peak areas for each standards pair using the pairwise matrix error formula ($\sigma_{std\;1,std\;2})$ described in the Methods section

The purpose of this study was to (i) develop and apply a quantitative approach for estimating the extent of Compound × Individual interactions during HS-SPME analysis and (ii) examine the appropriateness of using a single surrogate standard for volatile profiling in a given matrix as compared to more accurate (and more tedious and expensive) methods such as recovery spikes and isotopically labeled standards. In brief, the approach is as follows:

1. Samples are spiked with a cocktail of internal standards prior to SPME-GC-MS analysis (Figure 1). In the present work, as we were studying plant populations, these standards were either isotopic analogues of plant-derived odorants, or non-labeled surrogate standards previously reported for use in plant volatile profiling.
2. Pairwise matrix error ($\sigma_{std\;1,std\;2}$) is calculated as described in the Methods section and Figure 1, where the Compound × Individual interaction is assessed across a population for each internal standard, and quantified by the $\sigma_{std\;1,std\;2}$ value.

As proof of principle of the approach, we evaluated two plant populations where genome sequencing has been performed and for which there is interest in understanding the biochemical pathways responsible for regulating plant health, i.e., resistance to biotic and abiotic stress, as well as fruit quality [26]: (i) a *Vitis* spp. grape population (“Horizon” × Illinois 547-1) that had recently been genotyped using next-generation sequencing (NGS) approaches [7,27], and (ii) a tomato RIL population, which was recently genotyped by using the GBS (genotyping by sequencing) method [28] and evaluated using a single internal standard (Gonda et al., in preparation). While this approach yielded good results in a melon population [29], it is possible to discover better QTLs with better-matched internal standards.

### 2.2. Quantitating Compound × Individual Matrix Effects in a Grape Population

**Figure 2:**
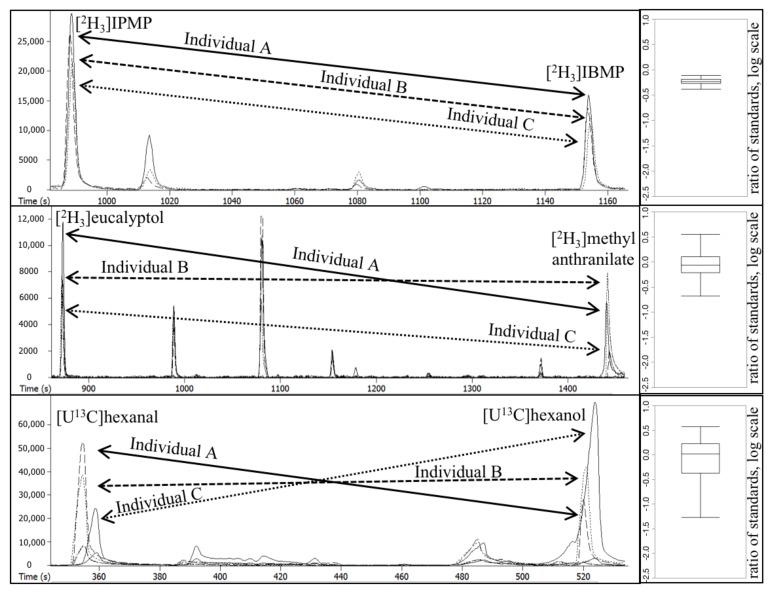
Chromatograms (left) and corresponding box plots (right) of Compound × Individual matrix effects for three pairs of internal standards in three different grape individuals (A, B, C). Internal standards are [2H3]IBMP and [2H3]IPMP (top), [2H3]eucalyptol and [2H3]methyl anthranilate (middle), and [U^13^C]hexanal and [U^13^C]hexanol (bottom).

In initial inspection of our HS-SPME-GC-MS dataset, we observed good reproducibility of internal standard peak areas for analytical replicates from the same grape individual ($\sigma_{x,within}$); precisions ranged over RSD = 12–20% for all volatiles except for [U^13^C]hexanal = 32% (data not shown). This range for precision is comparable to those in previous literature reports using HS-SPME-GC-MS on grape volatiles [30]. The presence of Compound × Individual interactions in the grape population is illustrated by three representative chromatograms, each depicting the behavior of a different compound pair across three individuals (Figure 2). For certain compound pairs, the variation in matrix effects is well correlated, e.g., for [2H3]IBMP and [2H3]IPMP, Figure 2 (top). Although the signal is ~50% higher in Individual C than in Individual A for [2H3]IPMP, a similar change is seen for [2H3]IBMP; thus, the two compounds could serve as surrogate standards. In contrast, the relative responses of other compound pairs ([2H3]eucalyptol and [2H3]methyl anthranilate, Figure 2 (middle); and [U^13^C]hexanal and [U^13^C]hexanol, Figure 2 (bottom)) were not consistent. For example, the [2H3]methyl anthranilate signal was 4-fold higher in Individual A than in Individual B, but the [2H3]eucalyptol signal was nearly unchanged. 

In our analyses, samples were prepared and run in batches of up to 30 runs. Since a run length was approximately 60 min, including oven cooling time, some samples would have sat for up to 30 hours before analysis. Although we used brine addition to disrupt enzymatic activity and randomized the order of analyses, we were still concerned that variation in the signal could have arisen from nonenzymatic reactions or residual enzymatic activity. To evaluate this possibility, regression analyses of peak area versus run number were performed for each standard. Representative plots for [U^13^C]hexanal and [U^13^C]hexanol are shown in Supplementary Figure S2; other plots are not shown. With the exception of [U^13^C]hexanal (*p* = 0.01, r^2^ = 0.18), no significant effect of run number was observed for any of the internal standards. The effect of run number on [U^13^C]hexanal was negative (decreasing signal intensity over time), and we observed that eliminating the first five vials of each batch from the ANOVA resulted in no significant correlation between run number and signal (*p* > 0.05, data not shown). The higher [U^13^C]hexanal response in earlier runs was not due to instability of the standard under aqueous conditions—we observed no change in [U^13^C]hexanal in a model juice system over 48 h (data not shown), nor was an increase observed for [U^13^C]hexanol during later runs (Supplementary Figure S2). An alternate explanation for the higher [U^13^C]hexanal in the first few runs of each batch is that the compound reacted with other nucleophilic juice components (e.g., polyphenols) [31]. Regardless of the cause, the effect of pre-analysis time explained only a small portion of the total variation observed in the [U^13^C]hexanal response among individuals.

**Figure 3:**
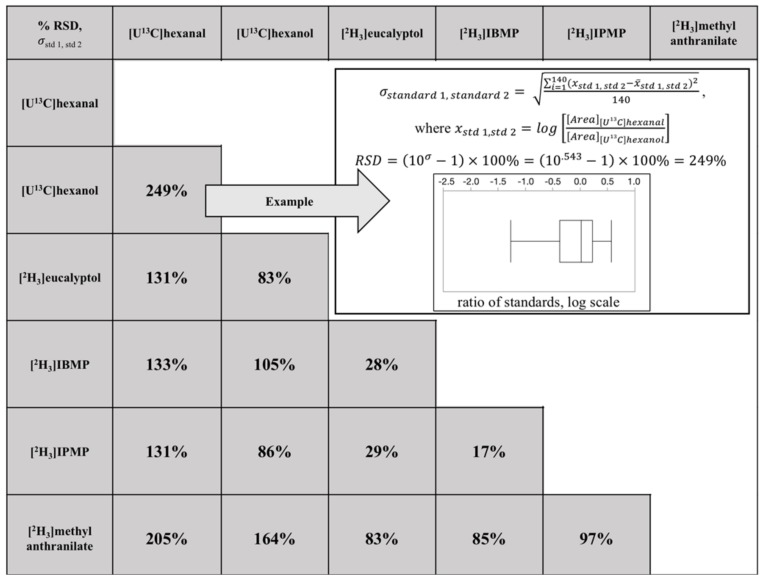
Compound × Individual matrix effects (%RSD) for pairs of six isotopically labeled internal standards in a grape population.

To quantitate Compound × Individual matrix effects ($\sigma_{std\;1,std\;2}$) we used the approach described in the previous subsection and outlined in Figure 1. Summary statistics are shown for each compound pair (Figure 3), where the percent relative standard deviation was calculated from the log-normalized pairwise matrix error. The pairwise matrix error for any two compounds will approach zero assuming that the compounds have minimal Compound × Individual matrix effects. Pairwise errors ranged from 17% to 249% for the 15 possible pairwise combinations. The smallest pairwise error (17%) was observed for [2H3]IPMP and [2H3]IBMP. This error is only modestly worse than precisions reported for 200 pg/g spikes of unlabeled IBMP and IPMP in Cabernet franc grape matrixes, quantified against [2H3]IBMP, which had % CVs of 2–8% [32]. IBMP and IPMP are homologues differing only by the presence of an additional -CH_2_- group in IBMP, and thus are expected to share similar chemical properties. Thus, it is unsurprising that the pairwise matrix error is small. However, the pairwise matrix error was considerably worse for most of the other labeled analyte pairs. For example, the RSD associated with [2H3]methyl anthranilate quantified by [2H3]eucalyptol, [2H3]IBMP, or [2H3]IPMP ranged from 83 to 97%, while the minimum RSD associated with quantifying a compound by either [U^13^C]hexanal or [U^13^C]hexanol was 83% and rose to 249% when quantifying [U^13^C]hexanal with [U^13^C]hexanol. Interestingly certain combinations have low pairwise errors in spite of having different functional groups, e.g., [2H3]eucalyptol had low error (28–29%) when paired with either [2H3]IBMP or [2H3]IPMP. 

### 2.3. Quantifying Compound × Individual Matrix Effects in a Tomato RIL Population

**Figure 4:**
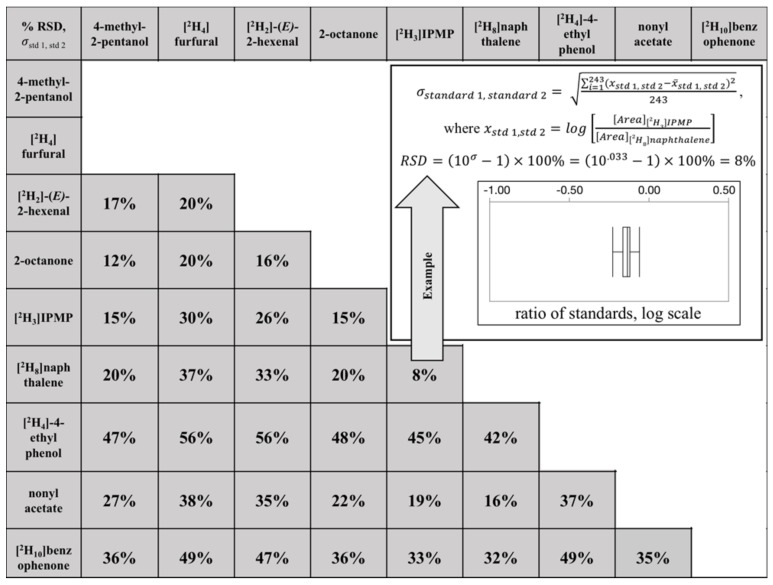
Compound × Individual matrix effects (%RSD) for pairs of nine non-native internal standards in a tomato population.

We trialed our approach with a tomato RIL population spiked with nine internal standards. This tomato population was selected because it had previously undergone volatile profiling using a single, non-native internal standard (2-octanone) [2]. The pairwise matrix error ranged from 8% to 56% among the 36 standard pairs evaluated (Figure 4). As with the grape population, compound pairs that showed the greatest differences in response across individuals (high values for $\sigma_{std\;1,std\;2}$) were not readily predictable from their chemistry. For example, it has been previously reported that volatiles with aromatic rings could participate in π-π interactions, decreasing their SPME response [33]. We therefore expected that internal standards with aromatic rings (e.g., [2H4]-4-ethyl phenol) should exhibit more correlated responses across individuals (lower $\sigma_{std\;1,std\;2}$ values) than with nonaromatic standards. However, we observed that [2H4]-4-ethyl phenol showed similar (and relatively high) pairwise variation with respect to both straight-chain compounds (2-octanone, nonyl acetate; $\sigma_{std\;1,std\;2}$ = 37–48%) and other aromatic compounds ([2H8]naphthalene, [2H10]benzophenone, [2H3]IPMP; $\sigma_{std\;1,std\;2}$ = 42–49%). Conversely, [2H3]IPMP showed similar matrix effects with both a mid-chain branched alcohol (4-methyl-2-pentanol; $\sigma_{std\;1,std\;2}$ = 15%) and the polyaromatic [2H8]naphthalene ($\sigma_{std\;1,std\;2}$ = 8%). Overall, Compound × Individual matrix effects in the tomato population were considerably less than those observed in the grape population, with $\sigma_{std\;1,std\;2}$ values ranging from 8% to 56%. Even at the extreme case of mismatched standards ([2H4]-4-ethylphenol vs [2H4]furfural or [2H2]-(*E*)-2-hexenal), the extent of the error ($\sigma_{std\;1,std\;2}$ = 56%) may still be tolerable for many studies. The reason why Compound × Individual matrix effects occur to a lesser extent in the tomato population as compared to the grape population in our study is unclear, and further research is needed to determine if this is a general phenomenon or specific to this population. 

### 2.4. Consequences of Compound × Individual Matrix Effects within Plant Populations 

The sample matrix is well known to affect HS-SPME recovery, either through competition on the SPME fiber, or through altering the volatility of analytes [5]. A less-appreciated problem is that HS-SPME matrix effects can show Compound × Individual interactions, which will affect the accuracy of even semiquantitative analyses (relative responses). Using our novel approach (Figure 1) we quantitated the extent of such interactions; pairwise errors arising from Compound × Individual matrix effects ranged from 17% to 249% among the 15 standard pairs evaluated in a grape population (Figure 3) and from 8% to 56% among the 36 standard pairs in a tomato population (Figure 4). In the worst-case scenario for either population (RSD = 249%), the 95% confidence interval would extend over 2 orders of magnitude. In situations where large, qualitative variations in a trait are observed, these effects will likely be tolerable. For example, floral-smelling monoterpenes are up to 1000-fold higher in Muscat-type grapes as compared to non-Muscat grapes [12], and the trait is under the control of a single major locus (*VvDXS*). However, many volatiles in grapes (and other plants) vary over a more limited range. For example, IBMP concentrations are reported to range from <1 ng/L to 55 ng/L in wines produced from cultivars containing methoxypyrazines [34]. In these cases, a considerable portion of observed variation could arise from matrix effects rather than from real differences among samples, and the use of a poorly matched surrogate standard would likely obscure real differences. Although these issues could be addressed through approaches such as the use of isotopologues (stable isotope dilution analysis, SIDA), these standards are often expensive, challenging to synthesize, and/or not widely available [35]. Furthermore, the use of labeled standards requires that targets be identified prior to analysis, and therefore would not be appropriate for nontargeted studies in which analytes are identified post hoc. A key contribution of the approach described in this paper is that it allows for a quantitative estimate of the likely error associated with using a limited number of surrogate standards within a given matrix, and allows a researcher to determine if SIDA or other more involved approaches (e.g., recovery spikes) are advisable. Finally, although the work in this paper was limited to two plant populations, the approach should be broadly applicable to any study with many individual samples.

### 2.5. Conclusions

We have reported an approach to estimating the extent of Compound × Individual matrix effects during volatile analyses. In this approach, the variances of the ratios of non-native standard pairs are determined, and the range of these values establishes the error expected from using a single surrogate standard during volatile analyses. This report specifically focused on the use of HS-SPME-GC-MS for the characterization of volatiles in plant populations (tomato and grape), although the approach should be equally appropriate for application to other analytical techniques or populations. We observed much greater Compound × Individual matrix effects for compound pairs in the grape population, with RSD = 249% for the pair of hexanal and hexanol. We also observed that the best surrogate standard for a given compound could not be easily predicted from the chemical structures of the compound. Based on these results, in situations where surrogate standards are used in HS-SPME-GC-MS analyses, we recommend characterizing the extent of Compound × Individual matrix effects to confirm that these effects are small in comparison to the desired accuracy. 

## 3. Materials and Methods 

### 3.1. Chemical Reagents and Standards 

The following chemicals were purchased from Sigma-Aldrich (St. Louis, MO, USA): sodium phosphate mono- (≥99%) and di-basic (≥98%), methanol (≥99%; MeOH), ethylenediaminetetraacetic acid disodium salt dihydrate (EDTA; ≥99%), 2-octanone (≥98%), nonyl acetate (FCC), and 4-methyl-2-pentanol (≥98%). [2H3]eucalyptol (>95%; >99% isotopic purity), [2H3]methyl anthranilate (>95%; >99% isotopic purity), and [2H2]-(*E*)-2-hexenal (>90%; >99% isotopic purity) were purchased from aromaLAB (Planegg, Germany); [2H3]-3-isobutyl-2-methoxypyrazine (IBMP) (>98%; >99% isotopic purity), [2H3]-3-isoproyl-2-methoxypyrazine (IPMP) (>98%; >99% isotopic purity), [2H4]furfural (>99%; >99% isotopic purity), [2H8]naphthalene (>98%; >99% isotopic purity), and [2H4]4-ethyl phenol (99%; >98% isotopic purity) were purchased from C/D/N Isotopes (Pointe-Claire, QC, Canada); and [2H10]benzophenone (>99%; >99% isotopic purity) was purchased from o2si Smart Solutions (Charleston, SC). A [U^13^C] internal standard extract where the concentration of [U^13^C]hexanal and [U^13^C]hexanol was ca. 30 µg/mL in MeOH was prepared as described in Supplementary Information (Figure S1). Deionized distilled water (18 MΩ) was used for all experiments (EMD Millipore Advantage A10). For the grapes, a pH 7.0 buffer solution was prepared from 0.1 M sodium phosphate dibasic/0.1 M sodium phosphate monobasic. For the tomatoes, a pH 7.5 buffer solution was prepared from 0.1 M EDTA. Both were stored at 4 °C. 

### 3.2. Sample Collection of Grapes and Tomatoes

Grape samples for matrix effect evaluations were obtained from a research vineyard where seedlings from the cross of “Horizon” × Illinois 547-1 (*V. rupestris* × *V. cinerea*) [27] were grown. These vines were developed by Bruce Reisch of the Horticulture Section at the New York State Agricultural Experiment Station, Geneva, NY, USA. The population was planted in two phases (1991 and 1998) with 2.7 m spaces between rows and 1.2 m between vines. About 400 g of ripe berries were collected from each of the 140 progeny during the 2013 harvest. The bagged samples were transported on ice packs in coolers back to the research station, where they were immediately moved into −20 °C storage. 

Tomato fruit samples were obtained from an RIL population (148 lines) derived from an interspecific cross between *Solanum lycopersicum* L. breeding line NC EBR-1 and *Solanum pimpinellifolium* L. accession LA2093 [36]. Three plants of each of the 148 RILs and their two parents were grown in an open field in Live Oak, FL, USA during the spring of 2015. Red-ripe fruits were harvested from each plant, and pericarp tissues of at least three fruits per plant were flash-frozen in liquid nitrogen the following day. Samples were ground to a fine powder with an IKA A11 analytical mill (IKA^®^-Works, Inc., Wilmington, NC, USA) and stored in 50 mL centrifuge tubes at −80 °C for future analysis.

### 3.3. Sample Preparation of Grapes and Tomatoes

Standard sample processing approach for grapes: For each individual (*n* = 140), frozen berries were thawed for 10 min, and 150 g were destemmed and macerated for 60–90 s in a chilled 250 mL stainless steel Waring blender. Berry slurry (5 g per vial, done in duplicate) was immediately transferred to two amber 20 mL SPME vials prefilled with 3 g of NaCl. The pH 7 phosphate buffer (5 mL; 0.1 M) was added, along with 20 µL of [U^13^C] internal standard extract where the concentration of [U^13^C]hexanal and [U^13^C]hexanol was ca. 30 µg/mL in MeOH, as well as 20 µL of [2H3]eucalyptol, [2H3]methyl anthranilate [2H3]IBMP, and [2H3]IPMP, where concentrations were ca. 2 ng/mL, 20 ng/mL, 20 pg/mL, and 20 pg/mL, respectively.

Standard sample processing approach for tomatoes: Samples (*n* = 243, from 114 of the 148 lines) were prepared according to Tikunov et al. [37] with slight modifications. Briefly, 1.5 g of ground tomato fruit tissue was aliquoted from each 50 mL centrifuge tube into a precooled (dry ice) 15 mL centrifuge tube, and immediately placed back on dry ice and stored in −80 °C. Prior to the analysis, the samples were thawed in a 30 °C water bath for 2 min. Then, 1.5 mL of 100 mM EDTA solution was added to the 15 mL tube, and the tube was shaken vigorously. Subsequently, the slurry (~2 mL) was transferred to a 10 mL SPME vial containing 2.4 g CaCl_2_. The internal standard cocktail was immediately added. The cocktail contained 2-octanone, nonyl acetate, 4-methyl-2-pentanol, [2H2]-(*E*)-2-hexenal, [2H3]IPMP, [2H4]furfural, [2H8]naphthalene, [2H4]4-ethyl phenol, and [2H10]benzophenone; all concentrations were ca. 1.25 µg/mL. Samples were tightly capped, vortexed, and stored at 4 °C for 24 h prior to analysis. 

### 3.4. Analysis of Grape Volatiles by HS-SPME-GC-MS

Volatile quantification was performed via HS–SPME–GC–TOF–MS (Pegasus 4D, LECO Corp., St. Joseph, MI, USA). Although the instrument is capable of two-dimensional GC analyses, all work was carried out in 1-D GC mode with the modulator and secondary oven turned off. A 2 cm divinylbenzene/Carboxen^®^/polydimethylsiloxane (DVB/CAR/PDMS) fiber was used for all HS-SPME extractions, with an incubation temperature of 40 °C, a pre-extraction incubation time of 10 min, and 30 min for HS-SPME extraction. A split/splitless injector was used with a constant temperature of 250 °C. SPME injections were splitless with a flow rate of 50 mL/min and purge time of 3 min. Helium was used as a carrier gas at a flow rate of 1.5 mL/min. The GC column was a DB-5 ms (30 m × 0.25 mm × 0.25 μm, Varian, Walnut Creek, CA). The initial GC oven temperature was 50 °C and held for 5 min, then ramped to 180 °C at 5 °C per min, then ramped to 240 °C at 15 °C per min and held at 240 °C for 15 min. The TOF–MS was operated in EI mode with an ionization energy of 70 eV. The electron multiplier was set to 1700 V. MS data from *m*/*z* = 20−400 was stored at 5 Hz. Data processing was carried out by the LECO ChromaTOF software. The qualifier ions were as follows: for [U^13^C]hexanal, *m*/*z* 46, 60, 76, 88; for [U^13^C]hexanol, *m*/*z* 60, 74, 90; for [2H3]eucalyptol, *m*/*z* 114, 142, 157; for [2H3]methyl anthranilate, *m*/*z* 95, 122, 154; for [2H3]IBMP, *m*/*z* 127, 154, 169; and for [2H3]IPMP, *m*/*z* 127, 140, 155. The quantifier ions for [U^13^C]hexanal, [U^13^C]hexanol, [2H3]eucalyptol, [2H3]methyl anthranilate, [2H3]IBMP, and [2H3]IPMP were *m*/*z* 76, 74, 157, 154, 127, and 140, respectively. 

### 3.5. Analysis of Tomato Volatiles by HS-SPME-GC-MS

The same GC-TOF-MS instrument was used for tomato analyses. A 1 cm DVB/CAR/PDMS fiber was used for all HS-SPME extractions, with an incubation temperature of 50 °C, a pre-extraction incubation time of 5 min, and 30 min for HS-SPME extraction. A split/splitless injector was used with a constant temperature of 250 °C. SPME injections were splitless with a flow rate of 50 mL/min and purge time of 3 min. Helium was used as a carrier gas at a flow rate of 1 mL/min. The GC column was a CP-Sil 8 ms (30 m × 0.25 mm × 0.25 μm, Agilent, The Netherlands). The initial GC oven temperature was 45 °C and held 5 min, then ramped to 180 °C at 5 °C per min, then ramped to 280 °C at 25 °C per min and held at 280 °C for 5 min. The TOF-MS was operated in EI mode with an ionization energy of 70 eV. The electron multiplier was set to 1700 V. MS data from *m*/*z* = 41−250 was stored at 5 Hz. Data processing was carried out by the LECO ChromaTOF software. The qualifier ions were as follows: for 2-octanone, *m*/*z* 43, 58, 71, 128; for nonyl acetate, *m*/*z* 43, 56, 70, 98, 126; for 4-methyl-2-pentanol, *m*/*z* 45, 69, 87; for [2H2]-(*E*)-2-hexenal, *m*/*z* 57, 71, 85, 100; for [2H3]IPMP, *m*/*z* 127, 140, 155; for [2H4]furfural, *m*/*z* 42, 70, 98, 100; for [2H8]naphthalene, *m*/*z* 108, 136; for [2H4]4-ethyl phenol, *m*/*z* 111, 126; and for [2H10]benzophenone, *m*/*z* 82, 110, 192. The quantifier ions were as follows: for 2-octanone, *m*/*z* 128; for nonyl acetate, *m*/*z* 43; for 4-methyl-2-pentanol, *m*/*z* 45; for [2H2]-(*E*)-2-hexenal, *m*/*z* 85; for [2H3]IPMP, *m*/*z* 140; for [2H4]furfural, *m*/*z* 100; for [2H8]naphthalene, *m*/*z* 136; for [2H4]4-ethyl phenol, *m*/*z* 111; and for [2H10]benzophenone, *m*/*z* 110.

### 3.6. Statistical Analyses

Within- and across-replicate errors were calculated for each standard using the following formula, where $\sigma_{x}$ represents the standard deviation of the log transformed peak area:

(1) $$\sigma_{x}=\sqrt{\frac{\sum_{i\;=\;1}^{N}{\left(x_{i}-\bar{x}\right)}^{2}}{N}}$$

where $x_{i}=log\left[\frac{{\left[Area\right]}_{replicate\;1\;of\;individual\;i}}{\frac{{\left[Area\right]}_{replicate\;1}+\;{\left[Area\right]}_{replicate\;2}}{2}}\right]$ of the *i*th individual in the population for within-replicate error ($\sigma_{x,within}$), $x_{i}=\;log\left[\frac{{\left[Area\right]}_{replicate\;1\;of\;individual\;i}}{mean\;area\;of\;standard\;across\;sample\;population}\right]$ for across-replicate error ($\sigma_{x,\;across})$, and $\bar{x}$ = mean value of $x_{i}$ for the population. 

The pairwise matrix error ($\sigma_{standard\;1,standard\;2})$ for each pair of standards was calculated using the above formula for $\sigma_{x}$, where $x_{i}=log\left[\frac{{\left[Area\right]}_{standard\;1}}{{\left[Area\right]}_{standard\;2}}\right]$ and $\bar{x}$ = mean of the log-normalized ratios for each pairwise comparison (*n* = 140, *n* = 243). These comparisons were performed on all the internal standards.

The relative standard deviation (RSD) was calculated from the error by the following formula: $RSD=\left(10^{\sigma_{x}}-1\right)\times 100\%$ for $\sigma_{x,within}$, $\sigma_{x,\;across}$, and $\sigma_{\;standard\;1,standard\;2}$.

R Studio v 1.0.153 (R Studio, Boston, MA, USA) was used for statistical analysis; JMP v 12.0 (SAS Institute Inc., Cary, NC, USA) was used for data visualization.

## Supplementary Materials

The following are available online. Materials and methods for enzymatic synthesis of [U-^13^C]hexanal and [U-^13^C]hexanol via [U-^13^C]α-linoleic acid; Figure S1: Scheme for producing [U-^13^C]hexanal and [U-^13^C]hexanol; Figure S2: Plot of ordinal run number (i.e., sample queue assignment) versus log-normalized peak areas for [U13C]hexanal and [U13C]hexanol.
