# Supplementary material for: HS-SPME-GC-MS Analyses of Volatiles in Plant Populations—Quantitating Compound × Individual Matrix Effects
Source: Molecules. 2018 Sep 23;23(10):2436. doi: 10.3390/molecules23102436 (PMC6222754; doi:10.3390/molecules23102436)
Supplement: Supplementary file 1 [file molecules-23-02436-s001.pdf]

HS-SPME-GC-MS Analyses of Volatiles in Plant Populations – Quantitating Compound × Individual Matrix Effects

Elizabeth A. Burzynski-Chang,[1] Imelda Ryona,[1] Bruce I. Reisch,[2] Itay Gonda,[3] Majid R. Foolad,[4] James J. Giovannoni,[3] Gavin L. Sacks[1]*

[1] Department of Food Science, Stocking Hall, Cornell University, Ithaca, New York 14853; eab54@cornell.edu, ir45@cornell.edu, gls9@cornell.edu

[2] Horticulture Section, School of Integrative Plant Science, New York State Agricultural Experiment Station, Cornell University, Geneva, New York 14456; bir1@cornell.edu

[3] Boyce Thompson Institute for Plant Science, Ithaca, New York 14850; itaygonda@gmail.com, james.giovannoni@ars.usda.gov

[4] Department of Plant Science, Pennsylvania State University, University Park, Pennsylvania 16802; mrf5@psu.edu

* Correspondence: gls9@cornell.edu; Tel.: 1-607-255-2335

SUPPORTING INFORMATION

MATERIALS & METHODS

Chemical Reagents and Standards for [$U^{13}C$]Hexanal and [$U^{13}C$]Hexanol Synthesis. The following chemicals were purchased from Sigma-Aldrich (St. Louis, MO): linoleic acid (95%), [$U^{13}C$] α-linoleic acid (>97%; >98% 13C enrichment), Soybean lipoxygenase (LOX) (EC No. 1.13.11.12) type I-B (221700 units/mg), alcohol dehydrogenase (ADH) from *Saccharomyces cerevisiae* (15000 units/mg), β-Nicotinamide adenine dinucleotide (NADH), reduced disodium salt hydrate (>94%), hexanal (≥97%), and hexanol (≥98%). Chemicals for buffers (citric acid (≥99%), sodium phosphate mono- (≥99%), and di-basic (≥98%), sodium bicarbonate (≥99%), and sodium carbonate (≥99%)) and organic solvents – ethanol (≥98%; EtOH), methanol (≥99%; MeOH), dichloromethane (≥99%; DCM), and pentane (≥99%) – were also purchased from Sigma Aldrich.

Preparation of Stock and Working Solutions for [$U^{13}C$]Hexanal and [$U^{13}C$]Hexanol Synthesis

<u>Enzyme solutions:</u> Separate solutions of LOX (753780 units/mL) and ADH (39300 units/mL stock solution were prepared by addition to 20 mL of Milli-Q water. Each stock was then stored in glass vials at -80 °C in 1.5 mL aliquots and thawed prior to use.

<u>Chemical Standards:</u> A solution of linoleic acid (5% w/w) was prepared by weighing 0.5 g of linoleic acid into 9.5 g of EtOH. [$U^{13}C$]linoleic acid stock solution (0.1 g was diluted in EtOH solution yielding a 5.95% w/w stock solution. Unlabeled hexanal and hexanol were prepared in EtOH to yield 10 and 100 μg/mL working solutions. NADH stock solution was prepared by adding 20 mL of Milli-Q water into 1 g of NADH yielding a stock concentration of 0.05 g/mL.

Buffer solutions: pH 4.5, pH 7.0, and pH 9.5 buffer solutions were prepared from 0.1 M citric acid/0.2 M sodium phosphate dibasic, 0.1 M sodium phosphate dibasic /0.1 M sodium phosphate monobasic, and 0.1 M sodium bicarbonate/0.1 M sodium carbonate respectively. The solutions were stored at 3 °C.

Protocol for Enzymatic Synthesis of [U^{13}C]hexanal and [U^{13}C]hexanol from [U-^{13}C]α-linoleic acid

The protocol for generating [U^{13}C]hexanal and [U^{13}C]hexanol is shown in Figure S.1. The yield of hexanal and hexanol was determined by calibration against unlabeled standards on GC-MS.

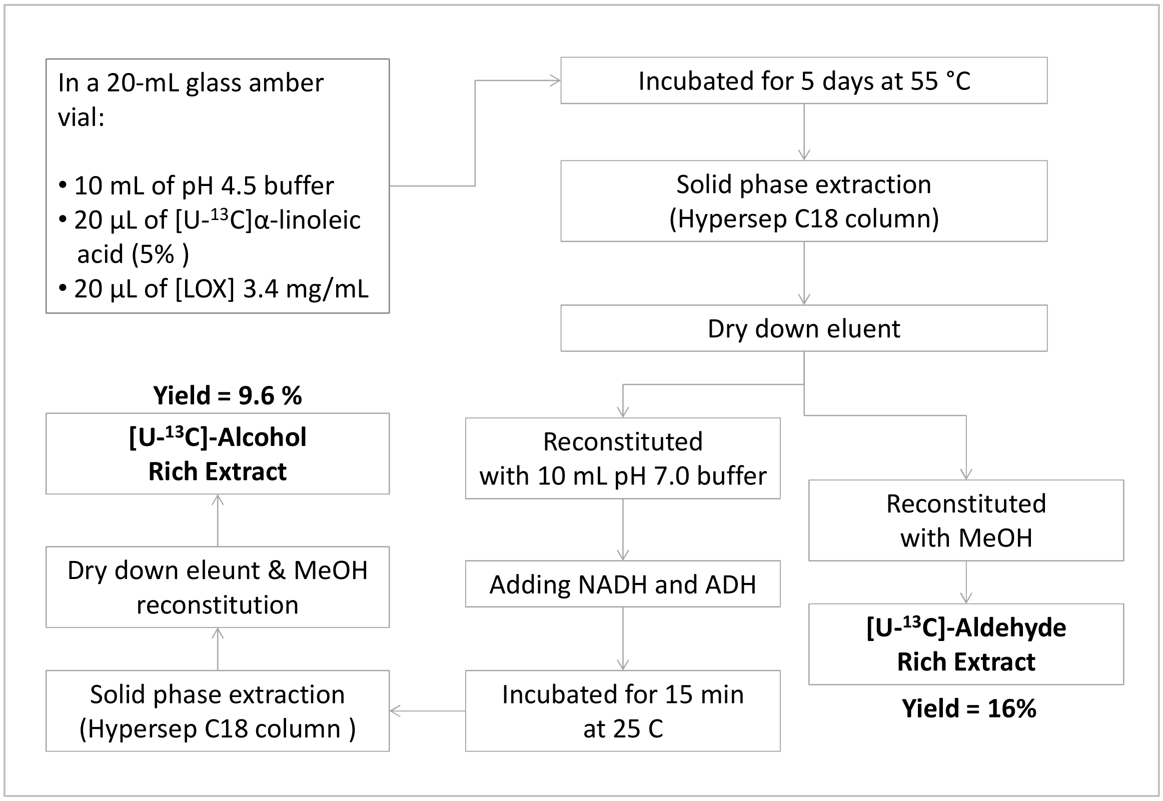

Figure S.1 – Protocol for generation of [U-^{13}C]hexanal and [U-^{13}C]hexanol from [U-^{13}C]α-linoleic acid.

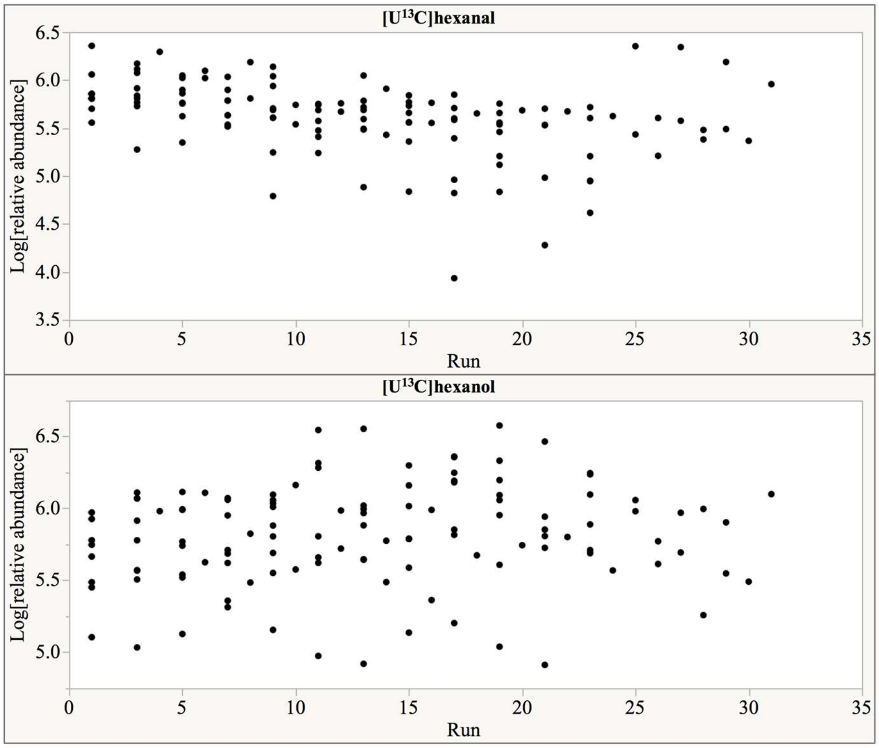

Figure S.2: Plot of ordinal run number (i.e. sample queue assignment) versus log-normalized peak areas for [U^{13}C]hexanal (top) and [U^{13}C]hexanol (bottom).
